# Laparoscopic Fertility Sparing Management of Cervical Cancer

**Published:** 2014-03-09

**Authors:** Chiara Facchini, Giuseppina Rapacchia, Giulia Montanari, Paolo Casadio, Gianluigi Pilu, Renato Seracchioli

**Affiliations:** 1 The Minimally Invasive Gynecological Surgery Unit, Department of Gynecology, S.Orsola-Malpighi Hospital, University of Bologna, Bologna, Italy; 2 Department of Obstetrics and Gynecology, S.Orsola-Malpighi Hospital, University of Bologna, Bologna, Italy

**Keywords:** Laparoscopy, Cervical Cancer, Cerclage, Pregnancy, Trachelectomy

## Abstract

Fertility can be preserved after conservative cervical surgery. We report on a 29-year-old woman who was obese, para 0, and diagnosed with cervical insufficiency at the first trimester of current pregnancy due to a previous trachelectomy. She underwent laparoscopic transabdominal cervical cerclage (LTCC) for cervical cancer. The surgery was successful and she was discharged two days later. The patient underwent a caesarean section at 38 weeks of gestation. Laparoscopic surgery is a minimally invasive approach associated with less pain and faster recovery, feasible even in obese women.

## Introduction

Worldwide, cervical cancer is the third diagnosed cancer and fourth leading cause of cancer deaths in women (1). It affects women of all age groups, including those in their fertility years. Due to the effective and widespread use of cervical carcinoma screening, many women could be diagnosed at early stage (2). The traditional primary management of early-stage cervical cancer is radical surgery. Dargent et al. (3) were the first to describe a radical vaginal excision of the cervix (radical trachelectomy) with pelvic node dissection to preserve the fertility. Appropriate criteria to propose a fertility-sparing surgery are as follows: i. a desire of future pregnancy, ii. a proven diagnosis of invasive cervical cancer, iii. squamous cell carcinoma, iv. adenocarcinoma or adenosquamous carcinoma, v. tumor size less than 2 cm, vi. International Federation of Gynecology and Obstetrics (FIGO) stage IA1 with lymphovascular space invasion (LVSI), and vii. FIGO stages IA2 and IB1cervical cancer (1). Successful pregnancy could occur after fertility-sparing surgery, although it may be complicated by cervical incompetence with higher risk of miscarriage, premature delivery, as well as neonatal morbidity and mortality (4). Cervical incompetence treatment consists of placing a pure string suture around the cervix, while the conventional method is by placing the suture vaginally. In 1965, Benson and Durfee (5) described a transabdominal laparotomy approach for women in whom a vaginal approach was deemed impossible. The reported success rate of abdominal cerclage is 85 to 90%. In recent years, the minimally-invasive approach has been introduced and there aren't clear differences between both methods in term of perinatal outcomes (6).

We present a case of a pregnant obese patient who previously underwent a fertility-sparing surgery.

## Case report

**Fig 1:**
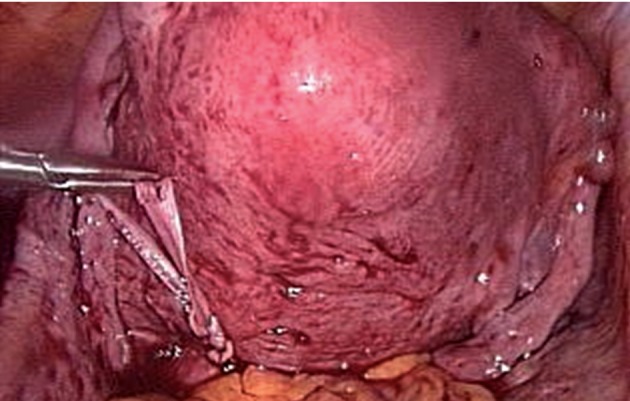
Laparoscopic view of the placement of the suture at posterior cervical isthmus.

**Fig 2:**
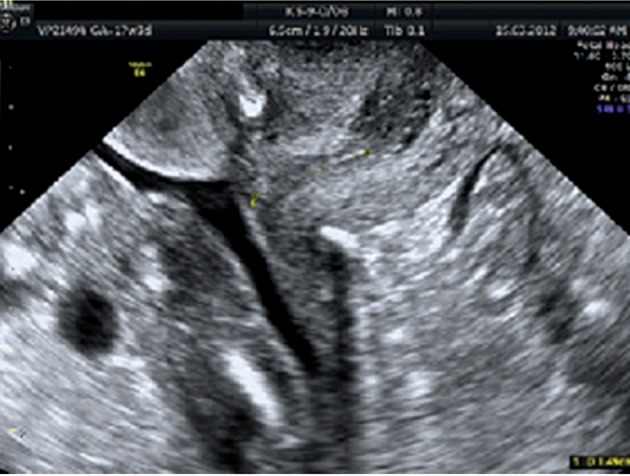
Cervical length with cerclage in situ by transvaginal ultrasound.

**Fig 3:**
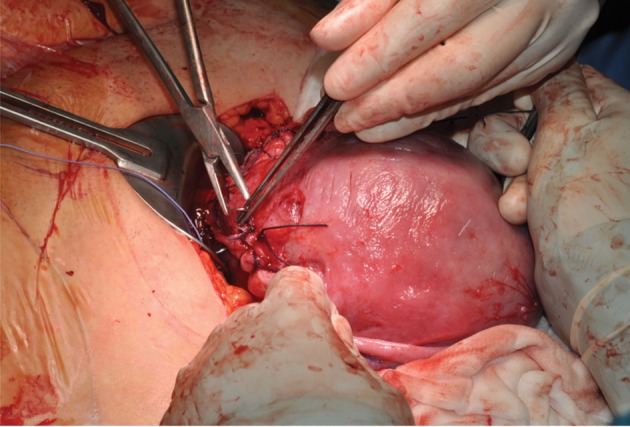
Uterine suture during caesarean section.

 A 29-year-old obese woman with body mass index (BMI) of 30.4, para 0, and large for gestational age (LGA) came to our clinic (S. Orsola-Malpighi Hospital, Bologna, Italy), in January 2012 for a short cervical length evaluation. She had a history of oncological surgery for squamocellular cervical cancer two years earlier (stage I (T1N0M0)). She underwent a vaginal trachelectomy and laparoscopic pelvic node dissection in our hospital (S. Orsola-Malpighi Hospital, Bologna, Italy). The follow-up examination was uneventful, and subsequently, she had a spontaneous conception. Transvaginal ultrasound scanning confirmed a single intrauterine pregnancy at 10 weeks of gestation. The size was consistent with dates and the result of nuchal translucency screening, performed at 11 weeks’ gestation, was normal. Her cervical length was <1 cm. Because of a virtually nonexistent cervix, we proposed a laparoscopic transabdominal cervical cerclage (LTCC) that was performed at 12 weeks' gestation. Under general anaesthesia, the patient was placed in dorsal lithotomy position and a Foley catheter was inserted. No vaginal instrumentation was used. The intervention was done with minimal uterine manipulation and minimal dissection. The vesico-uterine peritoneum was open and the bladder was dissected off the lower uterine segment bluntly. It was pierced the broad ligament medial to the uterine vessels with a laparoscopic suturing device without dissecting the uterine vessels. Mersilene band was placed at uterus at the level of cervical isthmus, and it was then knotted against the posterior cervical isthmus (Fig 1). Bladder integrity was preserved. The operation lasted 65 minutes. Fetal cardiac activity was confirmed before and after the procedure. The patient was discharged from the hospital 2 days later. The follow-up ultrasound during the rest of her pregnancy was uneventful (Fig 2). She underwent a caesarean section at 38 weeks of gestation because of the onset of labour (Fig 3). Intraoperative inspection of the surgical site revealed mature peritoneal tissue covering the tape, without adhesions. The cerclage tape was left in situ at the end of the caesarean section for future pregnancies. Birthweight was 3770 g with Apgar scores of 9 at both 1^st^ and 5th minutes and pH was 7.18. The mother and the baby were discharged 48 hours after.

## Discussion

Current management of early stage cervical cancer in most young women consists of radical hysterectomy or radiotherapy, both of which will inevitably compromise fertility (7). In 1994, Dargent et al. (3) described a group of patients who underwent a radical vaginal excision of cervix with laparoscopic pelvic node dissection, preserving the uterus. Now radical trachelectomy can be successfully performed in almost 50% of women with early-stage cervical cancer (8, 9) and oncologic outcomes are comparable to those of radical hysterectomy (1). Spontaneous pregnancy rate is almost 70% (10). Cerclage likely contributes to a post-trachelectomy uterine ability to carry a pregnancy to the third trimester (8).

Traditionally, it is performed vaginally, but there are cases that require an abdominal approach (6). Indications for abdominal cerclage are as follows: i. extremely short or absent cervix, ii. amputated cervix, iii. scarred cervix, and iv. previous failed vaginal cerclage (11). At the beginning, it was placed during laparotomy, but with advances in the field of minimally invasive surgery, laparoscopic technique has been recently presented (12). It has the same indications (13) and similar effectiveness (12). Moreover, laparoscopic cerclage offers the benefits of reduced postoperative pain, faster recovery and a success rate of 79-100% (6). It can be placed before pregnancy or at the end of the first trimester. 

In our case, both lymphoadenectomy and cerclage were performed by laparoscopy, even in obese patients. In the past, obesity was considered a relative contraindication to operative laparoscopy. Many currently available studies have demonstrated that laparoscopy is not only safe and practicable, but it achieves the same results as open technique. Moreover, as compared with the open procedure, the laparoscopic approach results in fewer operative complications, faster recovery and less need for pain medication. The decreased risk of adhesion formation is another major advantage of minimally invasive approach which may guarantee the achievement of a spontaneous future pregnancy.

Therefore, it should be particularly appropriate for obese women (14, 15). However, these assumptions are valid if these cases are managed in a tertiary care centre with multidisciplinary team of skilled specialists both in laparoscopic surgery and in the management of high risk pregnancy working together.

The main complications of laparoscopic cerclage are miscarriage and preterm labour. Other ones described are preterm premature ruptures of membrane, chorioamnionitis, and uterine rupture. Burger et al. (6) carried out a systematic review of literature about abdominal cerclage placed both laparoscopically and laparotomically. They analyzed percentage of these complications in the two groups and they didn’t find any difference between the two methods. 

Trachelectomy is a safe treatment to preserve fertility in selected women with early stage cervical cancer. Conception rate is high, but premature delivery caused by cervical insufficiency is common. Traditional treatment consists of placing a vaginal cerclage around cervix through an abdominal approach. Laparoscopic cerclage is a valid alternative to laparotomic procedure. It offers the benefits of reduced post-operative pain, faster recovery and fewer adhesions. It can be left in situ for women who desire a future pregnancy, while it is safe and feasible even in obese women.
